# Toxicity with small molecule and immunotherapy combinations in non-small cell lung cancer

**DOI:** 10.1007/s00262-020-02714-5

**Published:** 2020-09-11

**Authors:** H. Adderley, F. H. Blackhall, C. R. Lindsay

**Affiliations:** 1 Department of Medical Oncology, The Christie NHS Foundation Trust, Manchester, UK; 2 Division of Molecular and Clinical Cancer Sciences, University of Manchester, Manchester, UK; 3 Cancer Research UK Lung Cancer Centre of Excellence, London and Manchester, Manchester, UK

## Abstract

Treatment stratification in stage IV NSCLC is guided by identification of oncogene driver mutations. Actionable mutations with current licenced therapeutic agents include epidermal growth factor receptor (EGFR), rearrangements of anaplastic lymphoma kinase (ALK), ROS-1 and BRAF V600. Alongside progress with small molecule therapy, developments in immune checkpoint inhibitors (CPIs) have transformed the landscape of stage III and stage IV NSCLC. The success of CPIs has led to evaluation with small molecule therapy in both concurrent and sequential settings. In this review we summarise recent results of combination CPIs and tyrosine kinase inhibitors (TKIs) in stage IV NSCLC, detailing significant toxicity and its potential mechanisms with both concurrent and sequential approaches. As more therapeutic targets are being discovered it is becoming increasingly important for clinicians to correctly sequence therapy for delivery of safe and effective treatment. In addition to stage IV disease we suggest that comprehensive molecular profiling of key NSCLC drivers, particularly in stage III disease, will help to inform optimal treatment sequencing and minimise potential toxicity.

## Introduction

Molecular aberrations or oncogene driver mutations in non-small cell lung cancer (NSCLC) are frequently used to stratify treatment for patients with stage IV disease. Actionable mutations in the epidermal growth factor receptor (*EGFR*), rearrangements of anaplastic lymphoma kinase (*ALK*) *ROS*-*1* and *BRAF V600* represent targets with current licenced therapeutic agents that can improve progression-free survival (PFS) and overall survival (OS) [1, 2]. These include EMA- and FDA-approved erlotinib, gefitinib, afatinib and osimertinib for EGFR-mutant cancers. Alectinib, crizotinib, ceritinib, brigatinib and lorlatinib for ALK-rearranged cancers, as well as dabrafenib and trametinib for BRAF V600 mutant disease [3–15]. Other oncogene driver mutations or alterations are the subject of ongoing evaluation in clinical trials, with further standard of care breakthroughs expected for small molecules targeting NTRK, RET, HER2/MET and KRAS G12C subsets in particular. Indeed, the FDA have recently approved selpercatinib for RET and entrectinib for NTRK mutations [16, 17].

In parallel to this progress, recent developments in immune checkpoint inhibitors (CPIs) have transformed the landscape of stage III and IV NSCLC, conferring significant improvements in PFS and OS in the first and second line settings for advanced stage disease (KEYNOTE 024, OAK, KEYNOTE 010, CM017, CM057) [18–22], as well as improvements in PFS and OS when used as consolidation therapy after concurrent chemo-radiotherapy (CCRT) in stage III disease (PACIFIC) [23]. The success of CPIs has led to their investigation in combination with established therapies including chemotherapy, tyrosine kinase inhibitors (TKIs) and radiotherapy, with further advances in KEYNOTE-189, Keynote-407 and IMpower-130 establishing combination chemo-immunotherapy as a first line treatment option in advanced NSCLC lacking EGFR mutation or ALK rearrangement [24–26]. In addition, IMpower-150 demonstrated an overall survival signal benefit in a subgroup of patients with sensitising *EGFR* mutation with combination chemo-immunotherapy plus bevacizumab vs bevacizumab and chemotherapy alone. Based on these results, atezolizumab, bevacizumab, carboplatin and paclitaxel (ABCP) have been approved by the EMA as first-line therapy for stage IV non-squamous NSCLC including those with low or negative programmed death-ligand 1 (PD-L1) expression. This recommendation includes EGFR and ALK positive tumours who have failed previous targeted therapy [27, 28].

The past year has seen evaluation of CPIs in combinations with established TKIs for the first time, so far offering some reasons for disappointment. One particular emerging concern has been the development of severe toxicity, both with concurrent and sequential use of CPI/TKIs. Here we detail results of toxicity when CPIs are used concurrently with TKIs, and late CPI toxicity when CPIs and TKIs are used sequentially.

### CPIs in combination with TKIs

To date, there have been a number of phase I and II clinical trials across various molecular subgroups of NSCLC using different CPI/TKI combinations (Table 1). Toxicity concerns were identified in the TATTON trial [29] evaluating the combination of osimertinib, an EGFR TKI used in first line and acquired T790M resistance, in combination with durvalumab, a monoclonal antibody against PD-L1. The trial included two arms: (i) a dose escalation/expansion in EGFR-TKI pre-treated patients, and (ii) osimertinib/durvalumab in EGFR-TKI treatment naïve patients. Interstitial lung disease (ILD) was identified in 38% (13/34) of patients across treatment arms, 26% (6/23) in pre-treated patients and 64% (7/11) in treatment naïve patients. Five cases were reported as grade 3 or 4, most managed with steroids. Of 21 evaluable patients in the pre-treated cohort, 57% (12/21) had a partial response (PR) and 43% (9/21) stable disease (SD). Of 10 evaluable patients from the treatment-naïve arm, 80% (8/10) had PR and two had SD. Despite these efficacy results, investigators concluded that the primary end point of safety was not met and enrolment into the osimertinib and durvalumab treatment arm was terminated early. Enrolment into the expansion cohorts of MEK and MET inhibitor combinations demonstrated feasibility of combining osimertinib 80 mg with selumetinib or savolitinib. Similarly, a phase 1/2 study of crizotinib and nivolumab in ALK positive NSCLC was terminated prematurely due to safety concerns [30]. Five of the first 13 patients (38%) developed ≥ grade 3 hepatic dysfunction, of which 2 patients died, 38% (5/13) had a PR. The combination of nivolumab and ceritinib has been investigated by Felip and colleagues in a recent phase Ib multicentre study [31]. Dose limiting toxicities were identified in 17% of patients (6/36); most frequently reported grade 3/4 adverse events (AEs) were increased ALT (25%), increased GGT (22%) and rash (11%). Adverse events requiring dose change were reported in 33% (12/36) of patients and dose interruptions in 81% patients (29/36). Due to toxicity concerns, the trial was amended to allow alternative dosing strategy involving a run-in period of ceritinib followed by combination with nivolumab. Authors reported an overall response rate (ORR) of 68.8% and exploratory analysis indicated that PD-L1-positive disease was more likely to respond than PD-L1 negative, with overlapping confidence intervals.

**Table 1:** Safety of Concurrent CPI and TKI therapy

References	Phase	Setting	Oncogenic driver	No. patients	Arms/Treatment	Safety	Status
TATTON, [29]	Ib	1st line +	EGFR	34 Part A 23	Part A: 80 mg osimertinib od + durvalumab 3 mg/kg or 10 mg/kg IV q2w	ILD^a^ 38% (13/34)	Terminated early
				Part B 11	Part B: 80 mg osimertinib od + durvalumab 10 mg/kg IV q2w		
Group E checkmate 370, [30]	I/II	1st line	ALK	13	Nivolumab 240 mg q2w + crizotinib 250 mg bd	Hepatic toxicity 38% (5/13)	Terminated early
Felip E et al. [31]	Ib	1st line +	ALK	36 Group 1 14	Group 1: Nivolumab 3 mg/kg q2w + ceritinib 450 mg od	All grade diarrhoea 69% (25/36), all grade rash 64% (23/36)	Amended
				Group 2 22	Group 2: Nivolumab 3 mg/kg q2w + ceritinib 300 mg od		
Ma B et al. [32]	Ib	1st line	EGFR	28	Erlotinib 150 mg PO od + Atezolizumab 1200 mg q3w	AE 39% (11/28)	Completed
Gibbons DL et al. [33]	I	1st line	EGFR	20 Arm 1 10	Arm 1 Durvalumab 10 mg/kg q2w + gefitinib 250 mg od	AE 20% (4/20)	Completed
				Arm 2 10	Arm 2: 4 weeks of priming Gefitinib 250 mg od followed by Durvalumab 10 mg/kg q2w + gefitinib		
Gettingher et al. [34]	I	1st and 2nd line	EGFR	21 (1) Tx naive	Nivolumab 3 mg/kg q2w + erlotinib 150 mg od	AE 24% (5/21)	Completed
JAVELIN Lung 101, [35]	Ib	2nd line +	ALK	28	Avelumab 10 mg/kg q2w + Lorlatinib 100 mg od	AE 53.6% (15/28)	Completed
Kim DW et al. [36]	Ib	1st Line	ALK	21	Alectinib 600 mg bd + atezolizumab 1200 mg q3w	AE 52.4%	Completed

^a^*ILD* interstitial lung disease

In contrast to the safety concerns discussed above, other early phase clinical trials of concurrent CPI/TKI have shown acceptable safety profiles. Ma and colleagues evaluated the combination of erlotinib plus atezolizumab in *EGFR*-mutant disease [32]. Grade 3 or 4 AEs occurred in 39% (11/28) of patients, with pyrexia and increased ALT representing the most common AEs. No dose limiting toxicities were identified, a response rate (RR) of 75% and median PFS of 11.3 months was demonstrated in the expansion stage (*n* = 20). Another study evaluated durvalumab in combination with gefitinib in EGFR-mutant NSCLC, concluding that treatment was generally tolerable given a treatment discontinuation rate of 20% due to grade 3/4 toxicity (4/20 patients: increased ALT and/or AST in three, and pneumonitis in one). RR in 19 evaluable pts at 8 weeks was 78–80% [33]. Nivolumab and erlotinib was analysed by Gettingher and colleagues in first- and second-line therapy: grade 3 toxicity (increased liver enzymes two patients, diarrhoea two patients, weight loss one patient) occurred in 24% (5/21) of patients, with no grade 4/5 adverse events. In the TKI-treated group, RR was 15% and 24-week PFS was 48%. Authors concluded that this combination therapy was tolerable with durable response [34].

JAVELIN 101, a phase Ib study of concurrent avelumab and lorlatinib in ALK-positive patients, also demonstrated a reassuring safety profile, with no dose limiting toxicities [35]. Pre-treated patients received lorlatinib and avelumab. Despite a relatively high frequency of grade 3 and above toxicity, 53.6% (15/28), the side effect profile was manageable. The most commonly reported toxicities included hypertriglyceridemia (14.3%, *n* = 4) and GGT increase (10.7%, *n* = 3). A RR of 46.4% was demonstrated and results from the phase II study in treatment naïve patients are awaited. Kim and colleagues evaluated the combination of alectinib plus atezolizumab in ALK positive, treatment naïve patients; treatment related grade 3 adverse events were reported in 52.4% of patients. There were no dose limiting toxicities observed. At median follow up of 13 months, ORR was 81% (95% CI 58.1–94.6). Authors conclude that early efficacy results are encouraging and combination CPI plus TKI appears to be tolerable; however, as toxicities weren’t specified, one can only assume they were easy to manage [36].

In summary, although some promising efficacy was seen using concurrent CPI/TKI combinations, significant CPI-associated morbidity and mortality was a striking concern in many cases, leading to the early discontinuation of some studies. Ongoing studies include (NCT02323126) evaluating efficacy and safety of nivolumab in combination with EGF816, a third generation TKI, and nivolumab in combination with INC280 in those previously treated with EGF816. Results are awaited from the phase I/Ib study (NCT02039674), evaluating pembrolizumab in combination with afatinib after progression on erlotinib and the phase Ib study (NCT02013219), evaluating atezolizumab in combination with erlotinib or alectinib. Although it is very early to judge, no clear patterns have emerged from trials as yet to highlight specific drugs or targets of concern that may be vulnerable to these problems.

### Sequential CPIs and TKIs

As patients progress through treatment, a proportion will receive CPIs followed by TKIs. With durvalumab use as an adjuvant therapy following concurrent chemoradiotherapy (CCRT) in stage III NSCLC (PACIFIC trial), hazard ration (HR) for PFS for the whole study population was 0.55 (95% CI 0.45–0.68) in favour of durvalumab whereas, for EGFR-mutation positive patients, HR was not significant at 0.76 (95% CI 0.35–1.64) [23]. As molecular profiling is not performed routinely in all stage III NSCLC undergoing CCRT, there is, therefore, the potential for unidentified EGFR positive or ALK rearranged NSCLC patients to (i) receive limited benefit from the PACIFIC regimen, and (ii) be vulnerable to considerable toxicity if an actionable mutation is found at progression and sequential TKI therapy is employed. The reason for this latter concern is that, as well as side-effects with concurrent CPI/TKI combinations, recent retrospective reviews have highlighted a risk of serious toxicity when TKIs are prescribed sequentially following CPI use in stage III or stage IV disease.

Lisberg and colleagues evaluated pembrolizumab in a phase II clinical trial *of EGFR*-mutant TKI-naïve advanced NSCLC [37]. Recruitment was ceased due to lack of efficacy observed in 11 patients enrolled, with 1 patient demonstrating response later found to be EGFR wild type. Two deaths subsequently occurred within 6 months of enrolment, with one secondary to pneumonitis whilst on subsequent TKI. Of nine patients who received subsequent therapy, seven received erlotinib with a short median on-treatment duration of 109 days. AEs were secondary to TKI therapy in 86% (6/7) patients, with one case of grade 3 transaminitis and one grade 5 pneumonitis. The trial was not sufficiently powered to assess effects of prior pembrolizumab on subsequent EGFR TKI, although the results offered cause for concern regarding sequential use of CPI and TKI. Consistent with the above, Lin and colleagues have identified 11 patients with ALK fusion, ROS1 fusion or MET alteration that were treated in a single institution with CPI followed by crizotinib [38]. Hepatotoxicity with sequential CPI and TKI was significantly higher than that seen with TKI alone, including grade 3 or 4 increased ALT in 45.5% (5/11) vs. 8.1% (34/442 [95% CI 5.4–10.5, *p* < 0.0001]), and grade 3 or 4 increased AST in 36.4% (4/11) vs. 3.4% (14/442 [95% CI 1.9–5.5, *p* < 0.0001]). No grade 5 events occurred and all hepatotoxicity was reversible.

Another retrospective series analysed checkpoint inhibition followed by osimertinib [39]. In this study, severe CPI-related AEs were identified in 15% (6/41) of patients on osimertinib, with toxicity most common in those who had a shorter interval between CPI and TKI (< 3 months since CPI: 5 of 21 patients, 24%; > 3–12 months since CPI: 1 of 8 patients, 13%). AEs occurred at a median onset of 20 days after osimertinib (range 14–167 days), with all patients requiring steroids and most needing hospitalization. No toxicity was identified in those treated with sequential osimertinib followed by CPI (*n* = 29) or those treated with CPI followed by other EGFR-TKIs, afatinib or erlotinib, (*n* = 27).

In another observational study, sequential and combination nivolumab and EGFR-TKI therapy was evaluated [40]. 25.7% (18/70 [95% CI 16.0–37.6]) developed ILD. Stratification of patients by treatment with nivolumab revealed a strikingly high odds ratio of EGFR–TKI-associated ILD (5.09, 95% CI 2.87–9.03) compared to those who did not receive nivolumab 1.22 (95% CI 1.00–1.47); however, authors did not specify which EGFR-TKI was used in those who developed ILD. No other toxicities were evaluated. Amongst the 18 patients who developed ILD, the order of administration was identified in 15 patients all with nivolumab followed by TKI. The latency period from discontinuation of drug to development of ILD ranged from 19 to 147 days. Authors did not specify which EGFR-TKI was used with nivolumab but concluded that risk of EGFR-TKI ILD is increased with use of both CPI and TKI therapy. In contrast to previous toxicities described, early phase clinical trials have evaluated RET TKI BLU 667(NCT03037385) [41], poziotinib (NCT03066206) and TAK-788 (NCT02716116) in EGFR exon 20 ins mutation [42, 43]. Results have demonstrated efficacy without increased toxicity despite some patients having previous TKI and CPI. Ongoing trials are evaluating these biomarkers and targeted therapies. The mechanisms to explain synergistic toxicity of some sequential CPI and TKI therapy is poorly understood, yet allowing for limitations from the above retrospective series it may be that toxicity is drug rather than class specific with crizotinib and osimertinib causing toxicity as discussed.

The main sequence of sequential therapy evaluated has been immunotherapy followed by TKI. However, the ATLANTIC study did assess durvalumab after TKI in EGFR or ALK positive NSCLC, in the third line setting with an acceptable safety profile [44]. Of note, the cohort of patients (*n* = 77) with a sensitising *EGFR* mutation in IMpower-150 previously treated with TKI therapy did not appear to demonstrate increased toxicity. No grade 5 immune-related AEs occurred in the EGFR positive population [27]. Studies described in Tables 1, 2 have similarly failed to identified increased toxicity with prior TKI use suggesting that as the half-life of TKIs is shorter than immunotherapy this confers a potential lower risk of toxicity [29, 31, 34, 35, 39].

**Table 2:** Safety of sequential CPI/TKI and TKI/CPI therapy

References	Phase	Oncogenic driver	No. patients	Arms/Treatment	Safety	Status
Lin et al. [38]	Retrospective	ALK (3), ROS-1 (3), MET (5)	11	Pembrolizumab followed by crizotinib (6) Nivolumab followed by crizotinib (3) Atezolizumab followed by crizotinib (1) Nivolumab + ipilimumab followed by crizotinib (1)	G3/4 increase ALT 45.5% (5/11) G3/4 increase AST 36.4% (4/11)	NA
Schoenfeld et al. [39]	Retrospective	EGFR	41	Nivolumab followed by osimertinib (24) Pembrolizumab followed by osimertinib (9) Atezolizumab followed by osimertinib (8)	AE 15% (6/41) G3 pneumonitis (n = 4), G3 colitis (*n* = 1), G4 hepatitis (*n* = 1)	NA
Oshima et al. [40]	Retrospective	EGFR	70	Nivolumab followed TKI	ILD 25.7% (18/70)	NA
Garassino et al. [44]	II	EGFR	111	TKIs followed by Durvalumab	G3/4 AE 5% (6/111), G3 pneumonitis (*n* = 1)	Ongoing

### Discussion and conclusions

Here we have detailed past and present studies which highlight an emerging concern regarding serious toxicity of CPIs in combination with TKIs, whether prescribed concurrently or sequentially for stage IV NSCLC. As yet it is very early to state if there are clear patterns across these studies which may implicate certain drugs, targets or dosing schedules. Time from previous CPI appears to be a factor that can influence sequential TKI toxicity, perhaps reflecting the long half-life of CPIs and a context where we sometimes observe late immune-related toxicities months beyond treatment completion. Given the questionable efficacy of CPIs in never smoking subsets typically treated with TKIs, these challenges offer further reasons to be cautious in development of immunotherapy for this population. While most practice-changing NSCLC CPI studies have tended to exclude EGFR and ALK positive patients, a subgroup analysis of the IMpower-150 trial stands alone in describing benefit for EGFR-mutant patients to a CPI-based regimen [27].

The mechanism of toxicity with combination or sequential CPI and TKI therapy is poorly understood. CPIs release T cells from malignant immune suppression which could lead to generalised off-target immune system activation. It is proposed that TKIs may lead to release of tumour associated antigens via tumour cell death, increasing both basal and IFN gamma induced major histocompatibility complex (MHC) class-I presentation which in turn react with T cells causing heightened toxicity. TKIs may, therefore, harbour underappreciated immunomodulatory effects. More research is needed to establish what is truly going on [45, 46].

As more therapeutic targets are being discovered, it is becoming increasingly important for clinicians to correctly sequence CPIs and TKIs for delivery of safe and effective treatment. The results described above suggest that comprehensive molecular profiling of key NSCLC drivers, including in stage III disease, will help to inform optimal treatment sequencing and minimise potential toxicity. Future scrutiny will also involve CPI combination with TKI in never smoking molecular subsets beyond EGFR and ALK positive cancers, including those with BRAF V600, RET fusion and MET exon 14 who have shown promising TKI efficacy [47–49].
